# The general public’s perspectives on telemedicine during the COVID-19 pandemic in Korea: analysis of a nationwide survey

**DOI:** 10.4178/epih.e2022020

**Published:** 2022-02-04

**Authors:** EunKyo Kang, Hyejin Lee, Ki Jeong Hong, Jieun Yun, Jin Yong Lee, Yun-Chul Hong

**Affiliations:** 1 National Cancer Control Institute, National Cancer Center, Goyang, Korea; 2 Department of Family Medicine, National Cancer Center, Goyang, Korea; 3 Department of Family Medicine, Seoul National University Bundang Hospital, Seongnam, Korea; 4 Department of Emergency Medicine, Seoul National University Hospital, Seoul, Korea; 5 Department of Pharmaceutical Engineering, Cheongju University, Cheongju, Korea; 6 ublic Healthcare Center, Seoul National University Hospital, Seoul, Korea; 7 HIRA Research Institute, Health Insurance Review and Assessment Service, Wonju, Korea; 8 Department of Preventive Medicine, Seoul National University College of Medicine, Seoul, Korea; 9 Institute of Environmental Medicine, Seoul National University Medical Research Center, Seoul, Korea

**Keywords:** COVID-19, Telemedicine, Awareness, Participation, Intention

## Abstract

**OBJECTIVES:**

We investigated the awareness, experience, approval, intention to use, and the desired type of telemedicine among Korean general public.

**METHODS:**

From November to December 2020, we conducted an online self-reported survey on awareness, experience, approval, and intent to use telemedicine services among Korean residents aged 20 years or older. A total of 2,097 participants completed the survey.

**RESULTS:**

Of the 2,097 participants, 1,558 (74.3%) were aware of, 1,198 (57.1%) approved of, and 1,474 (70.3%) had the intention to use telemedicine. Participants from regions other than the Seoul metropolitan area and Daegu–Gyeongbuk Province (adjusted odds ratio [aOR], 1.29; 95% confidence interval [CI], 1.02 to 1.63), households with a monthly household income of US$6,000 or more (aOR, 1.44; 95% CI, 1.01 to 2.08), participants who had a college/university or associate’s degree (aOR, 1.35. 95% CI, 1.04 to 1.75) or a master’s degree or above (aOR, 1.73; 95% CI, 1.20 to 2.50), and housewives (aOR, 1.30; 95% CI, 1.03 to 1.64) had higher odds of approval. Elderly participants, those with a chronic disease (aOR, 1.26; 95% CI, 1.04 to 1.54), those who had experienced delays of healthcare services (aOR, 1.94; 95% CI, 1.27 to 2.96), and those who had experience with telemedicine (aOR, 4.28; 95% CI, 1.69 to 10.82) were more likely to intend to use telemedicine services. Regarding types of telemedicine, teleconsultation between doctors showed the highest approval rate (73.1%).

**CONCLUSIONS:**

In the context of the coronavirus disease 2019 pandemic, more than 70% of participants had already used or intended to use telemedicine at some point. Groups with a substantial need for telemedicine were more in favor of telemedicine.

## INTRODUCTION

Following the World Health Organization’s pandemic declaration in March 2020, the coronavirus disease 2019 (COVID-19) outbreak has resulted in an unprecedented global health crisis [1-3]. Social distancing became the global norm in order to prevent the spread of this novel disease. In addition, as a suboptimal substitute for in-person care, telemedicine has been recommended for patients with both chronic and acute diseases to prevent the transmission of the virus [4]. In Korea, telemedicine first emerged in Daegu and Gyeongsangbuk Province, which were most strongly affected by COVID-19 in the early stages of the outbreak.

Globally, telemedicine services have emerged as an alternative to in-person care that does not interfere with the continuity or quality of care. Before the pandemic, telemedicine improved access to care for those in underserved areas who experienced difficulty accessing healthcare [5,6]. Previous studies have reported that telemedicine positively affected the prevention, evaluation, management, and monitoring of disease [7] and reduced healthcare costs due to a decrease in emergency room visits and hospitalizations [8]. Patients who received telemedicine services reported satisfaction with the overall services, most specifically in regards to communication with healthcare professionals, cost-effectiveness, and time savings [9]. Healthcare professionals have also reported that telemedicine is advantageous for communicating with patients, as it is cost-effective and time-effective. However, despite these benefits for expanding healthcare access, there are concerns about health inequities among vulnerable populations who are in a lower socioeconomic status and less likely to have access to the necessary technology and knowledge of how to use it [10,11].

It has been argued that the telemedicine system, which was efficiently built during the COVID-19 pandemic, should be actively utilized even after COVID-19 [12,13]. Telemedicine, from the doctor’s perspective, has desirable outcomes, such as efficiency and cost-effectiveness of care. However, there has been limited research from patients’ and the general population’s perspectives towards telemedicine; thus, demands for telemedicine, attitudes towards it, and the desired type of telemedicine are not fully understood. In Korea, changes in perspectives towards telemedicine may occur, since telemedicine has been temporarily allowed during the pandemic. The aim of this study is to investigate the awareness, experience, approval, and intention to use telemedicine, as well as the perceived reasonable cost range and the desired type of telemedicine, in a nationwide sample.

## MATERIALS AND METHODS

### Data collection and recruitment

We performed a cross-sectional study using survey data (n=2,097) from November 10 to December 4, 2020. The survey candidates were selected using stratified sampling by age, sex, and geographical region. Those who were informed of the purpose of the study and consented to participate were enrolled in the study.

### Measurements

The online survey consisted of a set of questions on basic demographics, self-reported changes in health status during the COVID-19 pandemic, chronic disease management, awareness of telemedicine, and attitudes toward telemedicine. In the survey, telemedicine was defined as “the remote consultation and prescription to patients over a wired/wireless phone and video without a direct visit to healthcare providers when doing so is deemed to be safe according to the physician’s judgment.” The demographic variables included age, sex, area of residence, household income, education level, supplementary private insurance, marital status, and occupation. Participants were categorized into 5 age groups (20-29, 30-39, 40-49, 50-59, and 60 and older), and 3 groups in terms of the area of residence (Seoul metropolitan area, Daegu-Gyeongbuk Province, and others), which were categorized according to the magnitude of the COVID-19 outbreak. Monthly household income (US$) was divided into 4 groups (≤ 2,000, 2,000-3,999, 4,000-5,999, ≥ 6,000), and education level was divided into 3 groups (high school graduate and under, college/university graduate or associate’s degree, and master’s degree or above). Marital status was categorized into 3 groups (single, married, and divorced or widowed), and occupations were divided into 4 groups (office workers, manual workers, self-employed, and housewife/student/unemployed). Self-reported health status before and after the COVID-19 pandemic was measured on a 5-point Likert scale, and then divided into 3 groups (unchanged, improved, or worsened) [14,15]. The questions regarding chronic disease management asked whether participants currently had a chronic disease (yes/no); then, those who did were further asked whether they have experienced delays in healthcare services after the COVID-19 outbreak, for either chronic disease management (“Have you ever experienced delays in consultation or had problems while you needed a prescription refill or consultation resulting from an uncontrolled chronic disease?”) or any other conditions (“Have you ever experienced delays or disruptions in healthcare services, such as consultations, tests, and treatment?”).

Lastly, participants were asked about their awareness, experience, approval, and intent to use telemedicine services. They were asked to identify a reasonable range of cost as well as other factors important for telemedicine use. Regarding awareness, participants were asked to indicate whether they had heard of telemedicine. Those who indicated that they were aware of telemedicine were further asked whether they had experience with telemedicine. Then, a yes-or-no question inquired about participants’ feeling of approval of telemedicine after the COVID-19 pandemic. Participants were also asked to indicate whether they had a very low, low, high, or very high degree of intention to use telemedicine services. Additionally, participants were asked to choose their level of approval for each of the 6 types of telemedicine, which included (1) tele-consultations between doctors, (2) telemedicine between a doctor and a patient, (3) telemedicine between a doctor and a patient’s caregiver, (4) telemedicine for diagnosis or consultation (e.g. telepathology, teleradiology), (5) remote care in a ward or intensive care unit (e.g., a tele-intensive care unit), and (6) telemedicine in which a doctor continuously monitors a patient’s condition. Regarding the reasonable cost of telemedicine, participants were asked an open-ended question and answered with a specific amount. Lastly, regarding the factors to be considered for telemedicine, participants were asked, among a choice of 5, to select the factor with the highest priority. The choices included (1) the possibility of connecting to face-to-face treatment, if necessary, (2) availability whenever the patient needs, (3) management tailored to each patient’s situation and characteristics, (4) accessibility for use independent of disease type, and (5) availability without economic burden.

### Statistical analysis

We performed descriptive statistics and the chi-square test on demographics, awareness, experience, approval, intention to use, approval rate by type of telemedicine, and factors to be considered for telemedicine. We then performed multivariate logistic regression on factors associated with approval and intention to use telemedicine and the t-test or analysis of variance on the average reasonable copay amounts for telemedicine. Statistical significance was defined as a two-tailed p-value < 0.05. All statistical analyses were performed using Stata version 23 (StataCorp., College Station, TX, USA).

### Ethics statement

This study protocol was approved by the Seoul National University Hospital Institutional Review Board (IRB approval No. E-2011-102-1173).

## RESULTS

### Baseline characteristics of participants and people who had experience with telemedicine

Table 1 shows the basic demographics and awareness of telemedicine among the study participants. The study participants were evenly distributed by sex and across all age groups. Of the 2,097 participants, 401 (19.1%) lived in the Seoul metropolitan area, 196 (9.4%) lived in Daegu-Gyeongbuk Province, and 1,500 (71.5%) lived in other areas. The lowest household income group (below US$2,000) was the least frequent, with 192 (9.2%) participants. The majority of participants were college/university graduates or had associate’s degrees (n=1,498, 71.4%), and 1,718 (81.9%) held supplementary private health insurance. Married participants (n=1,251, 59.7%) and office workers comprised majorities (n=1,110, 52.9%). There were 1,081 (51.6%) participants with more than one pre-existing chronic condition. The majority of participants responded that their health had not changed (70.5%) after the COVID-19 pandemic. Furthermore, 108 (10.0%) participants reported delays in healthcare services for chronic diseases, while 159 (7.6%) reported delays for conditions other than chronic diseases. When we examined the characteristics of participants receiving telemedicine services among those who were aware of telemedicine (n=1,558), those who lived in other areas were less likely to have received telemedicine services than those who lived in the Seoul metropolitan area (3.3 vs. 7.9%, p=0.001). Participants with supplementary health insurance were more likely to receive telemedicine than those without (4.8 vs. 1.6%, p=0.03), while participants with pre-existing chronic disease were more likely to receive telemedicine than those without (6.3 vs. 1.8%, p<0.001). There were no statistically significant associations between receiving telemedicine services and household income, education level, marital status, and occupation (Table 1).

### Awareness, approval, and intention to use telemedicine

Of the 2,097 participants, 1,558 (74.3%) responded that they were aware of telemedicine. Older participants were more likely to be aware of telemedicine. All age groups showed a higher awareness rate than those 20-29 years old (p≤ 0.05). Participants with higher monthly household income, corresponding to US$4,000-5,999 (75.6 vs. 64.6%, p=0.003) and ≥ US$6,000 (78.8 vs. 64.6%, p<0.001), participants who had supplementary health insurance (75.7 vs. 67.8%, p=0.001), participants who were married (78.2 vs. 67.6%, p<0.001), and participants with underlying chronic diseases (78.9 vs. 69.4%, p<0.001) were more likely to be aware of telemedicine than their counterparts. Participants who had experienced delays in healthcare services for reasons other than chronic diseases were more likely to be aware of telemedicine than those who had not (85.5 vs. 73.4%, p=0.001).

With regard to approval, 1,198 (57.1%) of the total study population approved of telemedicine services. Females had a lower rate of approval than males (53.4 vs. 60.8%, p=0.001). All age groups showed a higher approval rate than those 20-29 years old (p≤0.020). Participants with a monthly income of more than US$6,000 (60.3 vs. 50.0%, p=0.010), participants with a master’s degree or above (63.8 vs. 54.0%, p=0.020), married participants (61.7 vs. 49.3%, p<0.001), self-employed participants (65.8 vs. 55.7%, p=0.009) had higher rates of approval of telemedicine. Participants with chronic diseases (60.8 vs. 53.3%, p=0.001), and participants who experienced delays in healthcare for conditions other than chronic diseases (66.0 vs. 56.4%, p=0.020) were more likely to approve telemedicine than their counterparts.

Among all participants, 1,474 (70.3%) intended to use telemedicine. Compared to those in their 20s, all other age groups were significantly more likely to use telemedicine (p≤ 0.03). Those without supplementary health insurance were less likely to use telemedicine treatment (62.5 vs. 72.0%, p<0.001). Married participants, (74.3 vs. 63.7%, p<0.001), those with chronic diseases (66.6 vs. 73.7%, p<0.001). Additionally, those who experienced delays in healthcare for a chronic disease after the COVID-19 outbreak (72.6 vs. 84.3%, p=0.010) or for any other conditions (69.4 vs. 818%, p=0.001), as well as those who had previously received telemedicine (72.6 vs. 92.5%, p=0.001) had more intent to use telemedicine (Table 2).

### Rate of approval according to the type of telemedicine

Among the types of telemedicine, teleconsultation between doctors (73.1%) had the highest rate of approval, followed by telemedicine for diagnosis or consultation, and telemedicine in which a doctor continuously monitors a patient’s condition. Telemedicine between a doctor and a patient’s caregiver (62.0%) had the lowest rate of approval. Each type of telemedicine showed differences with respect to socio-demographic factors, including age, household income, supplementary private insurance, marital status, and pre-existing chronic disease (Table 3).

### Factors associated with Intention to use telemedicine

When we analyzed the factors associated with approval of telemedicine, women had lower odds than men (adjusted odds ratio [aOR], 0.73; 95% confidence interval [CI], 0.61 to 0.88). Participants in their 60s had the highest odds (aOR, 2.59; 95% CI, 1.74 to 3.86). The aOR was higher in other regions (aOR, 1.29; 95% CI, 1.02 to 1.63), households with a household income of US$6,000 or more (aOR, 1.44; 95% CI, 1.01 to 2.08), participants with a college/university or associate’s degree (aOR, 1.35. 95% CI 1.04 to 1.75) or a master’s degree or above (aOR, 1.73; 95% CI, 1.20 to 2.50), and housewives (aOR, 1.30; 95% CI, 1.03 to 1.64). Those who experienced delays in treatment due to circumstances other than chronic diseases (aOR, 1.65; 95% CI, 1.16 to 2.35) and those who had experience with telemedicine (aOR, 1.16; 95% CI, 1.01 to 1.32) were also more likely to approve of telemedicine.

As for the factors associated with intent to use telemedicine, participants with older age had a higher aOR, while those without supplementary health insurance had a lower (aOR, 0.65; 95% CI, 0.51 to 0.84). Participants with a chronic disease (aOR, 1.26; 95% CI, 1.04 to 1.54), those who had experience delays of healthcare services (aOR, 1.94; 95% CI, 1.27 to 2.96), and those who had experience with telemedicine (aOR, 4.28; 95% CI, 1.69 to 10.82) were more likely to intend to use telemedicine services (Table 4).

### The appropriate amount of copay for telemedicine and factors considered to be important in telemedicine

The average reasonable copay amount for telemedicine was US$29.54. In particular, participants with chronic disease and those with improved health status after the COVID-19 pandemic were willing to pay higher rates for telemedicine (US$32.61, p=0.010 and US$49.49, p<0.001, respectively). Furthermore, participants who had experienced delays in healthcare services for both chronic diseases (US$75.03, p<0.001), and other conditions (US$51.01, p<0.001) were willing to pay more for telemedicine than other groups (Supplementary Material 1).

When participants were asked about factors considered to be important for telemedicine, the most frequently chosen was that management is tailored to each patient’s situation and characteristics (24.9%), followed by the possibility of connecting to face-to-face treatment if necessary (23.6%). Socio-demographic factors, including sex, monthly household income, education level, supplementary private insurance, and occupation, were associated with the prioritization of factors considered to be important for telemedicine (Supplementary Material 2).

## DISCUSSION

Telemedicine has contributed to the ability to continue delivering healthcare services under emergency circumstances in order to prevent the collapse of the health system [16]. The use of telemedicine to contain the spread of COVID-19 has become a global phenomenon [17,18]. Correspondingly, the Korean government has temporarily allowed teleconsultations starting on March 2, 2020 [19]. In this study, we investigated public opinion on telemedicine through a representative sample of the Korean population. We found that more than half of the total study population agreed with the implementation of telemedicine. Although telemedicine has been useful during emergency circumstances such as COVID-19 [20], several concerns remain that it is difficult to conduct direct consultations and complete lab tests. The main reported barriers to the implementation of telemedicine are insufficient understanding and access among users [21]. Furthermore, the sustainability of telemedicine in Korea is still controversial due to legal and ethical issues, as well as safety and responsibility among healthcare providers [22]. We explored these questions through the perspective of the public, as healthcare consumers, towards telemedicine, which will affect whether telemedicine will be implemented in the long term.

In this study, more than 70% of participants intended to use telemedicine, and this intention was associated with older age. Considering that the usage rate of telemedicine was higher among the younger age group [23], our study result suggests that older people have a high awareness of telemedicine and intention to use it, but there are barriers to actual use. Having additional private health insurance and a pre-existing chronic disease also showed a positive association with intention to use telemedicine. These findings aligned with previous studies that people with higher household income and chronic diseases were more likely to receive telemedicine services during the COVID-19 pandemic [23]. Similar to previous findings that people who received guidance on how to use the telemedicine platform and who had previously experienced telemedicine were more likely to approve of telemedicine, we found that those who previously used telemedicine were more in favor of the long-term use of telemedicine. In addition, participants who experienced delays in healthcare due to the COVID-19 outbreak were more likely to approve of telemedicine, which may reflect the need for remote care [24]. The main advantage felt by patients who have actually experienced telemedicine was convenience, and non-delayed care delivery and the benefits of receiving care in their own home were important factors for patients [25].

In regard to types of telemedicine services, teleconsultations between doctors had the highest approval rate, followed by teleradiology or telepathology. These results were different from those of previous studies, according to which patients agreed most with routine doctor visits, followed by post-surgery visits, expert consultations, and surgical remote mentoring in previous studies [26]. Teleconsultation between doctors was occasionally used in practice prior to the pandemic because it promoted access to healthcare in rural areas and increased the capacity of primary healthcare physicians [27,28], and a report found that medical staff working at private hospitals experienced fewer restrictions or barriers to telemedicine than medical staff working at university hospitals [29]. Thus, there was already a certain level of social acceptance for teleconsultation prior to its widespread use during the pandemic. Telemedicine between a doctor and a patient’s caregiver had the lowest approval rate, which may reflect the anxiety of the public regarding non-face-to-face care through a patient’s caregiver without direct patient contact.

In this study, the factor considered to be most important among study participants was management tailored to each patient’s situation and characteristics. According to Loeb et al. [30], selecting appropriate patients for telemedicine should be included in the task checklist for telemedicine launch. Likewise, it is remarkably important not only that appropriate patients should be selected for telemedicine services, but that the type of telemedicine should be tailored to each individual [31]. Previous studies that dealt with the advantages and disadvantages of telemedicine found that the main advantage was a reduction in travel and associated costs. Next, tailored care should be considered important, considering that physical examinations are limited in telemedicine. The possibility of connecting to face-to-face treatment if necessary is also an important factor related to previously reported limitations. The factor rated important by the fewest participants was accessibility for use independent of disease type, reflecting that patient-specific characteristics were considered more significant than disease-specific characteristics in telemedicine.

There are several limitations of this study. First, since the study population was limited to those speaking Korean and residing in Korea, our results may have limited generalizability to other populations. Second, although the study participants were recruited by stratifying the Korean population by age, sex, and region, selection bias may have occurred because they were given the option to participate in this study.

In conclusion, the COVID-19 pandemic has marked a turning point for not only healthcare providers, but patients and society. When we analyzed the survey results, it was found that the majority of the public was in favor of the use of telemedicine even after COVID-19. Interestingly, the approval of telemedicine had a positive correlation with age, indicating that technology use may not be a barrier to using telemedicine. Individuals with healthcare needs, such as those with chronic diseases and experiences of delays in healthcare services due to COVID-19, had a higher approval rate of telemedicine. Additionally, the financial status of patients (e.g., having supplementary health insurance) may potentially affect the approval of telemedicine. The general population considered individually tailored management to be important. Aspects for ensuring safety in care should be also considered while building infrastructure for telemedicine services after the COVID-19 pandemic. Telemedicine has demonstrated advantages in delivering timely care while minimizing exposure to COVID-19 and protecting healthcare providers and patients amid the COVID-19 pandemic, and it may be widely utilized after the pandemic.

## Figures and Tables

**Table 1.** Baseline characteristics of the study participants

Characteristics	Total	Experienced	p-value
Total	2,097 (100)	67 (4.3)	
Sex			
Male	1,058 (50.5)	37 (4.7)	
Female	1,039 (49.6)	30 (3.9)	0.450
Age (yr)			
20-29	377 (18.0)	10 (4.4)	
30-39	411 (19.6)	14 (5.0)	0.720
40-49	485 (23.1)	18 (5.0)	0.710
50-59	479 (22.8)	13 (3.2)	0.470
≥60	345 (16.5)	12 (4.2)	0.920
Region			
Seoul metropolitan area	401 (19.1)	23 (7.9)	
Daegu–Gyeongbuk Province	196 (9.4)	7 (4.8)	0.240
Others	1,500 (71.5)	37 (3.3)	0.001
Household income (US$)			
<2,000	192 (9.2)	4 (3.2)	
2,000-3,999	684 (32.8)	26 (5.3)	0.340
4,000-5,999	610 (29.2)	16 (3.5)	0.890
≥6,000	600 (28.8)	21 (4.4)	0.550
Educational status			
High school graduate and under	359 (17.1)	7 (2.7)	
College/university graduate	1,498 (71.4)	51 (4.6)	0.190
Master's degree or above	240 (11.4)	9 (5.0)	0.220
Private insurance			
Yes	1,718 (81.9)	63 (4.8)	
No	379 (18.1)	4 (1.6)	0.030
Marital status			
Single	755 (36.0)	17 (3.3)	
Married	1,251 (59.7)	48 (4.9)	0.160
Widowed/divorced	91 (4.3)	2 (2.9)	0.830
Job			
Office worker	1,110 (52.9)	40 (4.8)	
Manual worker	212 (10.1)	8 (5.4)	0.760
Own business	193 (9.2)	7 (4.5)	0.860
Housewife/Student/Unemployed	582 (27.8)	12 (2.9)	0.110
Having a chronic illness			
No	1,016 (48.5)	13 (1.8)	
Yes	1,081 (51.6)	54 (6.3)	<0.001
Subjective change in health status			
No change	1,478 (70.5)	32 (3.0)	
Improved	199 (9.5)	13 (8.3)	0.001
Worsened	420 (20.0)	22 (6.9)	0.002
Delayed treatment for chronic conditions			
No	973 (90.0)	22 (2.9)	
Yes	108 (10.0)	32 (34.4)	<0.001
Delayed elective treatment and treatment for non-chronic conditions			
No	1,938 (92.4)	33 (2.3)	
Yes	159 (7.6)	34 (25.0)	<0.001

Values are presented as number (%).

**Table 2.** Awareness, approval, and intention to use telemedicine

Variables	Awareness	p-value	Approval	p-value	Intention to use	p-value
Total	1,558 (74.3)		1,198 (57.1)		1,474 (70.3)	
Sex						
Male	790 (74.7)		643 (60.8)		757 (71.6)	
Female	768 (73.9)	0.690	555 (53.4)	0.001	717 (69.0)	0.200
Age (yr)						
20-29	230 (61.0)		169 (44.8)		226 (60.0)	
30-39	278 (67.6)	0.050	219 (53.3)	0.020	278 (67.6)	0.030
40-49	359 (74.0)	<0.001	263 (54.2)	0.006	342 (70.5)	0.001
50-59	403 (84.1)	<0.001	312 (65.1)	<0.001	348 (72.7)	<0.001
≥60	288 (83.5)	<0.001	235 (68.1)	<0.001	280 (81.2)	<0.001
Region						
Seoul metropolitan area	291 (72.6)		214 (53.4)		279 (69.6)	
Daegu-Gyeongbuk Province	145 (74.0)	0.720	109 (55.6)	0.610	137 (69.9)	0.940
Others	1,122 (74.8)	0.360	875 (58.3)	0.070	1,058 (70.5)	0.710
Household income (US$)						
<2,000	124 (64.6)		96 (50.0)		126 (65.6)	
2,000-3,999	491 (71.8)	0.060	386 (56.4)	0.110	483 (70.6)	0.190
4,000-5,999	461 (75.6)	0.003	352 (57.7)	0.060	428 (70.2)	0.240
≥6,000	473 (78.8)	<0.001	362 (60.3)	0.010	432 (72.0)	0.090
Educational status						
High school graduate and under	257 (71.6)		194 (54.0)		249 (69.4)	
College/university graduate	1,121 (74.8)	0.210	851 (56.8)	0.340	1,052 (70.2)	0.750
Master’s degree or above	180 (75.0)	0.360	153 (63.8)	0.020	173 (72.1)	0.470
Private insurance						
Yes	1,301 (75.7)		998 (58.1)		1,237 (72.0)	
No	257 (67.8)	0.001	200 (52.8)	0.060	237 (62.5)	<0.001
Marital status						
Single	510 (67.6)		372 (49.3)		481 (63.7)	
Married	978 (78.2)	<0.001	772 (61.7)	<0.001	929 (74.3)	<0.001
Widowed/divorced	70 (76.9)	0.070	54 (59.3)	0.070	64 (70.3)	0.210
Job						
Office worker	835 (75.2)		618 (55.7)		781 (70.4)	
Manual worker	149 (70.3)	0.130	125 (59.0)	0.380	158 (74.5)	0.220
Own business	157 (81.4)	0.070	127 (65.8)	0.009	144 (74.6)	0.230
Housewife/Student/Unemployed	417 (71.7)	0.110	328 (56.4)	0.790	391 (67.2)	0.180
Having a chronic illness						
No	705 (69.4)		541 (53.3)		677 (66.6)	
Yes	853 (78.9)	<0.001	657 (60.8)	0.001	797 (73.7)	<0.001
Subjective change in health status						
No change	1,084 (73.3)		843 (57.0)		1,042 (70.5)	
Improved	156 (78.4)	0.130	117 (58.8)	0.640	144 (72.4)	0.590
Worsened	318 (75.7)	0.330	238 (56.7)	0.890	288 (68.6)	0.470
Delayed treatment for chronic conditions						
No	760 (78.1)		592 (60.8)		706 (72.6)	
Yes	93 (86.1)	0.060	65 (60.2)	0.890	91 (84.3)	0.010
Delayed elective treatment and treatment for non-chronic conditions						
No	1,422 (73.4)		1,093 (56.4)		1344 (69.4)	
Yes	136 (85.5)	0.001	105 (66.0)	0.020	130 (81.8)	0.001
Experience with telemedicine						
No	-		965 (64.7)		1,083 (72.6)	
Yes	-		47 (70.2)	0.360	62 (92.5)	0.001

Values are presented as number (%).

**Table 3.** Approval rate according to the type of telemedicine

Variables		Teleconsultations between doctors	p-value	Telemedicine between a doctor and a patient	p-value	Telemedicine between a doctor and a patient's caregiver	p-value	Telemedicine for diagnosis or consultation	p-value	Remote care in a ward or ICU	p-value	Telemedicine in which the doctor continuously monitors the patient’s condition	p-value
Total		1,533 (73.1)		1,386 (66.1)		1,301 (62.0)		1,497 (71.4)		1,359 (64.8)		1,476 (70.4)	
Sex													
	Male	763 (72.1)		711 (67.2)		643 (60.8)		759 (71.7)		687 (64.9)		740 (69.9)	
	Female	770 (74.1)	0.300	675 (65.0)	0.280	658 (63.3)	0.230	738 (71.0)	0.720	672 (64.7)	0.900	736 (70.8)	0.650
Age (yr)													
	20-29	241 (63.9)		214 (56.8)		201 (53.3)		224 (59.4)		206 (54.6)		233 (61.8)	
	30-39	275 (66.9)	0.380	240 (58.4)	0.640	238 (57.9)	0.200	253 (61.6)	0.540	239 (58.2)	0.320	258 (62.8)	0.780
	40-49	365 (75.3)	<0.001	314 (64.7)	0.020	298 (61.4)	0.020	364 (75.1)	<0.001	319 (65.8)	0.001	341 (70.3)	0.009
	50-59	381 (79.5)	<0.001	352 (73.5)	<0.001	325 (67.9)	<0.001	375 (78.3)	<0.001	337 (70.4)	<0.001	374 (78.1)	<0.001
	≥60	271 (78.6)	<0.001	266 (77.1)	<0.001	239 (69.3)	<0.001	281 (81.5)	<0.001	258 (74.8)	<0.001	270 (78.3)	<0.001
Region													
	Seoul metropolitan area	310 (77.3)		269 (67.1)		259 (64.6)		302 (75.3)		256 (63.8)		298 (74.3)	
	Daegu–Gyeongbuk Province	138 (70.4)	0.070	129 (65.8)	0.760	116 (59.2)	0.200	133 (67.9)	0.060	129 (65.8)	0.640	139 (70.9)	0.380
	Others	1,085 (72.3)	0.050	988 (65.9)	0.650	926 (61.7)	0.300	1,062 (70.8)	0.080	974 (64.9)	0.680	1,039 (69.3)	0.050
Household income (US$)													
	<2,000	135 (70.3)		111 (57.8)		100 (52.1)		124 (64.6)		120 (62.5)		126 (65.6)	
	2,000-3,999	472 (69.0)	0.730	424 (62.0)	0.300	386 (56.4)	0.280	471 (68.9)	0.260	419 (61.3)	0.750	466 (68.1)	0.510
	4,000-5,999	466 (76.4)	0.090	427 (70.0)	0.002	402 (65.9)	0.001	450 (73.8)	0.010	396 (64.9)	0.540	434 (71.2)	0.150
	≥6,000	453 (75.5)	0.150	419 (69.8)	0.002	407 (67.8)	<0.001	445 (74.2)	0.010	417 (69.5)	0.070	444 (74.0)	0.030
Educational status													
	High school graduate and under	251 (69.9)		226 (63.0)		224 (62.4)		232 (64.6)		232 (64.6)		241 (67.1)	
	College/university graduate	1,109 (74.0)	0.110	998 (66.6)	0.190	930 (62.1)	0.910	1,093 (73.0)	0.002	961 (64.2)	0.870	1,061 (70.8)	0.170
	Master’s degree or above	173 (72.1)	0.570	162 (67.5)	0.250	147 (61.3)	0.780	172 (71.7)	0.070	166 (69.2)	0.250	174 (72.5)	0.160
Private insurance													
	Yes	1,276 (74.3)		1,159 (67.5)		1,098 (63.9)		1,246 (72.5)		1,131 (65.8)		1,217 (70.8)	
	No	257 (67.8)	0.010	227 (59.9)	0.005	203 (53.6)	<0.001	251 (66.2)	0.010	228 (60.2)	0.040	259 (68.3)	0.340
Marital status													
	Single	504 (66.8)		438 (58.0)		408 (54.0)		481 (63.7)		439 (58.2)		477 (63.2)	
	Married	958 (76.6)	<0.001	887 (70.9)	<0.001	834 (66.7)	<0.001	946 (75.6)	<0.001	859 (68.7)	<0.001	935 (74.7)	<0.001
	Widowed/divorced	71 (78.0)	0.030	61 (67.0)	0.100	59 (64.8)	0.050	70 (76.9)	0.010	61 (67.0)	0.110	64 (70.3)	0.180
Job													
	Office worker	803 (72.3)		736 (66.3)		689 (62.1)		789 (71.1)		700 (63.1)		785 (70.7)	
	Manual worker	154 (72.6)	0.930	134 (63.2)	0.380	141 (66.5)	0.220	153 (72.2)	0.750	148 (69.8)	0.060	141 (66.5)	0.220
	Own business	147 (76.2)	0.270	131 (67.9)	0.670	111 (57.5)	0.230	147 (76.2)	0.150	130 (67.4)	0.250	136 (70.5)	0.940
	Housewife/Student/Unemployed	429 (73.7)	0.550	385 (66.2)	0.950	360 (61.9)	0.930	408 (70.1)	0.670	381 (65.5)	0.330	414 (71.1)	0.860
Having a chronic illness													
	No	707 (69.6)		632 (62.2)		606 (59.7)		706 (69.5)		631 (62.1)		691 (68.0)	
	Yes	826 (76.4)	<0.001	754 (69.8)	<0.001	695 (64.3)	0.030	791 (73.2)	0.060	728 (67.4)	0.010	785 (72.6)	0.020
Subjective change in health status													
	No change	1,067 (72.2)		973 (65.8)		903 (61.1)		1,058 (71.6)		945 (63.9)		1,049 (71.0)	
	Improved	142 (71.4)	0.810	121 (60.8)	0.160	130 (65.3)	0.250	134 (67.3)	0.220	132 (66.3)	0.510	130 (65.3)	0.100
	Worsened	324 (77.1)	0.040	292 (69.5)	0.160	268 (63.8)	0.310	305 (72.6)	0.680	282 (67.1)	0.230	297 (70.7)	0.920
Delayed treatment for chronic conditions													
	No	1,170 (78.5)		1,059 (71.0)		992 (66.5)		1,136 (76.2)		1,008 (67.6)		1,124 (75.4)	
	Yes	54 (80.6)	0.680	47 (70.2)	0.880	40 (59.7)	0.250	52 (77.6)	0.790	46 (68.7)	0.860	45 (67.2)	0.130
Delayed elective treatment and treatment for non-chronic conditions													
	No	748 (76.9)		682 (70.1)		630 (64.8)		718 (73.8)		654 (67.2)		714 (73.4)	
	Yes	78 (72.2)	0.280	72 (66.7)	0.460	65 (60.2)	0.350	73 (67.6)	0.170	74 (68.5)	0.780	71 (65.7)	0.090
Experience with telemedicine													
	No	1,409 (72.7)		1,268 (65.4)		1,192 (61.5)		1,371 (70.7)		1,254 (64.7)		1,363 (70.3)	
	Yes	124 (78.0)	0.150	118 (74.2)	0.030	109 (68.6)	0.080	126 (79.3)	0.020	105 (66.0)	0.740	113 (71.1)	0.840

Values are presented as number (%).

**Table 4.** Factors affecting approval and intention to use telemedicine^1^

Variables	Approval of telemedicine	Intention to use telemedicine
Sex		
Male	1.00 (reference)	1.00 (reference)
Female	0.73 (0.61, 0.88)	0.93 (0.76, 1.13)
Age (yr)		
20-29	1.00 (reference)	1.00 (reference)
30-39	1.46 (1.07, 1.99)	1.29 (0.93, 1.77)
40-49	1.44 (1.03, 2.00)	1.42 (1.00, 2.01)
50-59	2.28 (1.60, 3.24)	1.63 (1.13, 2.36)
≥60	2.59 (1.74, 3.86)	2.68 (1.73, 4.14)
Region		
Seoul metropolitan area	1.00 (reference)	1.00 (reference)
Daegu–Gyeongbuk Province	1.12 (0.78, 1.59)	1.01 (0.69, 1.47)
Others	1.29 (1.02, 1.63)	1.05 (0.82, 1.35)
Household income (US$)		
<2,000	1.00 (reference)	1.00 (reference)
2,000-3,999	1.21 (0.86, 1.71)	1.07 (0.74, 1.54)
4,000-5,999	1.26 (0.88, 1.82)	1.00 (0.68, 1.46)
≥6,000	1.44 (1.01, 2.08)	1.08 (0.73, 1.59)
Educational status		
High school graduate and under	1.00 (reference)	1.00 (reference)
College/university graduate	1.35 (1.04, 1.75)	1.24 (0.94, 1.64)
Master's degree or above	1.73 (1.20, 2.50)	1.26 (0.85, 1.86)
Private insurance		
Yes	1.00 (reference)	1.00 (reference)
No	0.80 (0.62, 1.02)	0.65 (0.51, 0.84)
Marital status		
Single	1.00 (reference)	1.00 (reference)
Married	1.04 (0.81, 1.35)	1.08 (0.82, 1.42)
Widowed/divorced	1.03 (0.63, 1.69)	0.86 (0.51, 1.47)
Job		
Office worker	1.00 (reference)	1.00 (reference)
Manual worker	1.20 (0.87, 1.67)	1.24 (0.86, 1.78)
Own business	1.33 (0.95, 1.87)	1.11 (0.77, 1.61)
Housewife/Student/Unemployed	1.30 (1.03, 1.64)	0.97 (0.76, 1.25)
Having a chronic illness		
No	1.00 (reference)	1.00 (reference)
Yes	1.20 (1.00, 1.44)	1.26 (1.04, 1.54)
Subjective change in health status^2^		
No change	1.00 (reference)	1.00 (reference)
Improved	1.21 (0.88, 1.66)	1.17 (0.83, 1.65)
Worsened	1.06 (0.84, 1.33)	0.95 (0.75, 1.22)
Delayed treatment for chronic conditions^3^		
No	1.00 (reference)	1.00 (reference)
Yes	1.14 (0.74, 1.74)	2.04 (1.17, 3.54)
Delayed elective treatment and treatment for non-chronic conditions^4^		
No	1.00 (reference)	1.00 (reference)
Yes	1.65 (1.16, 2.35)	1.94 (1.27, 2.96)
Experience with telemedicine^5^		
No	1.00 (reference)	1.00 (reference)
Yes	1.16 (1.01, 1.32)	4.28 (1.69, 10.82)

Values are presented as adjusted odds ratio (95% confidence interval).

1 Adjustment for sex, age, region, household income, educational status, private insurance, marital status, job status, and having a chronic illness.

2 Adjustment for sex, age, region, household income, educational status, private insurance, marital status, job status, having a chronic illness, and experience with telemedicine.

3 Adjustment for sex, age, region, household income, educational status, private insurance, marital status, job status, and change in health status.

4 Adjustment for sex, age, region, household income, educational status, private insurance, marital status, job status, having a chronic illness, change in health status, and experience with telemedicine.

5 Adjustment for sex, age, region, household income, educational status, private insurance, marital status, job status, having a chronic illness, change in health status, and experience with telemedicine.

## SUPPLEMENTARY MATERIALS

Supplementary Material 1. Responses on the appropriate amount of copays for telemedicine

Supplementary Material 2 Factors considered to be important in telemedicine
