# Supplementary material for: The general public’s perspectives on telemedicine during the COVID-19 pandemic in Korea: analysis of a nationwide survey
Source: Epidemiol Health. 2022 Feb 4;44:e2022020. doi: 10.4178/epih.e2022020 (PMC9117104; doi:10.4178/epih.e2022020)
Supplement: Supplementary Material 1. — Responses on the appropriate amount of copays for telemedicine [file epih-44-e2022020-suppl1.docx]

Supplementary Material 1. Responses on the appropriate amount of copays for telemedicine

| Variable | | Appropriate amount of copays for telemedicine, US dollars (95% CI) | *P* value |
| --- | --- | --- | --- |
| Total | | 29.54 (27.06 - 32.02) |  |
| Sex | Men | 31.37 (27.50 - 35.25) | 0.14 |
|  | Women | 27.67 (24.59 - 30.76) |  |
| Age | 20-29 | 35.95 (28.37 - 43.53) | 0.03 |
|  | 30-39 | 30.64 (24.66 - 36.61) |  |
|  | 40-49 | 26.33 (22.05 - 30.61) |  |
|  | 50-59 | 24.54 (20.46 - 28.62) |  |
|  | ≥ 60 | 32.68 (26.33 - 39.03) |  |
| Region | Seoul metropolitan area | 36.03 (28.95 - 43.10) | 0.04 |
|  | Daegu–Gyeongbuk Province | 25.71 (19.63 - 31.78) |  |
|  | Others | 28.31 (25.51 - 31.10) |  |
| Household income | ≤$2,000 | 27.79 (18.61 - 36.97) | 0.10 |
|  | $2,000-3,999 | 31.85 (27.01 - 36.70) |  |
|  | $4,000-5,999 | 24.94 (21.89 - 28.00) |  |
|  | ≥$6,000 | 32.19 (27.05 - 37.32) |  |
| Educational status | High school graduate and under | 32.91 (26.37 - 39.46) | 0.10 |
|  | College / university graduate | 27.86 (25.14 - 30.58) |  |
|  | Master's degree or above | 34.98 (25.69 - 44.28) |  |
| Private insurance | Yes | 29.02 (26.51 - 31.52) | 0.38 |
|  | No | 31.92 (24.19 - 39.64) |  |
| Marital status | Single | 32.70 (27.79 - 37.61) | 0.11 |
|  | Married | 27.35 (24.73 - 29.98) |  |
|  | Widowed/divorced | 33.43 (15.55 - 51.30) |  |
| Job | Office worker | 29.94 (26.54 - 33.34) | 0.27 |
|  | Manual worker | 35.80 (26.56 - 45.04) |  |
|  | Own business | 28.97 (21.59 - 36.35) |  |
|  | Housewife/Student/Unemployed | 26.69 (22.14 - 31.24) |  |
| Having a chronic illness | No | 26.27 (23.32 - 29.23) | 0.01 |
|  | Yes | 32.61 (28.69 - 36.54) |  |
| Subjective change in health status | No change | 26.14 (23.79 - 28.49) | < 0.001 |
|  | Getting better | 49.49 (34.31 - 64.67) |  |
|  | Worsening | 32.06 (26.38 - 37.74) |  |
| Delayed treatment for chronic conditions | No | 27.91 (24.47 - 31.34) | < 0.001 |
|  | Yes | 75.03 (51.97 - 98.09) |  |
| Delayed elective treatment and treatment for non-chronic conditions | No | 27.78 (25.43 - 30.12) | < 0.001 |
|  | Yes | 51.01 (35.30 - 66.71) |  |
| Experience with telemedicine | No | 24.93 (22.41 - 27.44) | < 0.001 |
|  | Yes | 82.93 (52.27 - 113.58) |  |
