# Supplementary material for: The general public’s perspectives on telemedicine during the COVID-19 pandemic in Korea: analysis of a nationwide survey
Source: Epidemiol Health. 2022 Feb 4;44:e2022020. doi: 10.4178/epih.e2022020 (PMC9117104; doi:10.4178/epih.e2022020)
Supplement: Supplementary Material 2 — Factors considered to be important in telemedicine [file epih-44-e2022020-suppl2.docx]

Supplementary Material 2. Factors considered to be important in telemedicine

| Variable | Selection rate as priority, percent (95% CI) | | | | |  |
| --- | --- | --- | --- | --- | --- | --- |
|  | Possibility of connecting to face-to-face treatment if necessary | Availability whenever the patient needs | Management tailored to each patient's situation and characteristics | Accessibility for use independent of disease type | Availability without economic burden | *P* value |
| Total | 23.6 (21.8 - 25.4) | 19.6 (17.9 - 21.3) | 24.9 (23.0 - 26.7) | 9.0 (7.7 - 10.2) | 22.4 (20.6 - 24.2) |  |
| Sex |  |  |  |  |  |  |
| Men | 20.9 (18.4 - 23.3) | 22.5 (20.0 - 25.0) | 23.2 (20.6 - 25.7) | 9.0 (7.3 - 10.7) | 24.0 (21.4 - 26.6) |  |
| Women | 26.4 (23.7 - 29.1) | 16.7 (14.4 - 18.9) | 26.7 (24.0 - 29.3) | 9.0 (7.2 - 10.7) | 20.8 (18.3 - 23.3) | < 0.001 |
| Age |  |  |  |  |  |  |
| 20-29 | 25.7 (21.3 - 30.2) | 18.0 (14.1 - 21.9) | 25.2 (20.8 - 29.6) | 10.1 (7.0 - 13.1) | 19.9 (15.8 - 23.9) |  |
| 30-39 | 26.8 (22.5 - 31.1) | 22.4 (18.3 - 26.4) | 21.9 (17.9 - 25.9) | 9.0 (6.2 - 11.8) | 19.2 (15.4 - 23.0) | 0.40 |
| 40-49 | 22.1 (18.4 - 25.8) | 19.4 (15.9 - 22.9) | 24.7 (20.9 - 28.6) | 9.3 (6.7 - 11.9) | 24.3 (20.5 - 28.2) | 0.16 |
| 50-59 | 22.5 (18.8 - 26.3) | 16.7 (13.3 - 20.1) | 27.1 (23.1 - 31.1) | 8.1 (5.7 - 10.6) | 25.3 (21.4 - 29.2) | 0.10 |
| ≥60 | 21.2 (16.8 - 25.5) | 22.3 (17.9 - 26.7) | 25.2 (20.6 - 29.8) | 8.4 (5.5 - 11.3) | 22.3 (17.9 - 26.7) | 0.45 |
| Region |  |  |  |  |  |  |
| Seoul metropolitan area | 24.4 (20.2 - 28.7) | 18.7 (14.9 - 22.5) | 25.9 (21.6 - 30.2) | 9.5 (6.6 - 12.4) | 21.4 (17.4 - 25.5) |  |
| Daegu–Gyeongbuk Province | 19.9 (14.3 - 25.5) | 16.3 (11.1 - 21.5) | 25.5 (19.4 - 31.7) | 10.2 (5.9 - 14.5) | 27.0 (20.8 - 33.3) | 0.07 |
| Others | 23.9 (21.7 - 26.0) | 20.3 (18.2 - 22.3) | 24.5 (22.4 - 26.7) | 8.7 (7.2 - 10.1) | 22.1 (20.0 - 24.2) | 0.99 |
| Household income |  |  |  |  |  |  |
| ≤$2,000 | 19.3 (13.6 - 24.9) | 13.5 (8.7 - 18.4) | 24.0 (17.9 - 30.1) | 11.5 (6.9 - 16.0) | 31.8 (25.1 - 38.4) |  |
| $2,000-3,999 | 24.3 (21.1 - 27.5) | 18.7 (15.8 - 21.6) | 23.2 (20.1 - 26.4) | 9.1 (6.9 - 11.2) | 24.0 (20.8 - 27.2) | 0.005 |
| $4,000-5,999 | 23.1 (19.8 - 26.5) | 18.4 (15.3 - 21.4) | 27.5 (24.0 - 31.1) | 8.5 (6.3 - 10.7) | 22.0 (18.7 - 25.3) | 0.004 |
| ≥$6,000 | 24.8 (21.4 - 28.3) | 23.5 (20.1 - 26.9) | 24.5 (21.0 - 28.0) | 8.5 (6.2 - 10.7) | 18.2 (15.1 - 21.3) | < 0.001 |

Supplementary Material 2. Factors to be considered important in telemedicine (continued)

| Variable | Selection rate as priority, percent (95%CI) | | | | |  |
| --- | --- | --- | --- | --- | --- | --- |
|  | Possibility of connecting to face-to-face treatment if necessary | Availability whenever the patient needs | Management tailored to each patient's situation and characteristics | Accessibility for use independent of disease type | Availability without economic burden | *P* value |
| Educational status |  |  |  |  |  |  |
| High school graduate and under | 20.6 (16.4 - 24.8) | 17.0 (13.1 - 20.9) | 25.6 (21.1 - 30.2) | 9.7 (6.7 - 12.8) | 26.2 (21.6 - 30.8) |  |
| College / university graduate or associate’s degree | 23.7 (21.5 - 25.9) | 20.3 (18.3 - 22.3) | 25.2 (23.0 - 27.4) | 8.9 (7.5 - 10.4) | 21.5 (19.4 - 23.6) | 0.02 |
| Master's degree or above | 27.5 (21.8 - 33.2) | 19.2 (14.2 - 24.2) | 22.1 (16.8 - 27.4) | 7.9 (4.5 - 11.4) | 22.5 (17.2 - 27.8) | 0.04 |
| Private insurance |  |  |  |  |  |  |
| Yes | 24.0 (22.0 - 26.0) | 20.5 (18.6 - 22.4) | 24.9 (22.8 - 26.9) | 8.8 (7.5 - 10.2) | 21.5 (19.5 - 23.4) |  |
| No | 21.9 (17.7 - 26.1) | 15.6 (11.9 - 19.2) | 25.1 (20.7 - 29.4) | 9.5 (6.5 - 12.5) | 26.6 (22.2 - 31.1) | 0.02 |
| Marital status |  |  |  |  |  |  |
| Single | 24.6 (21.6 - 27.7) | 18.0 (15.3 - 20.8) | 23.3 (20.3 - 26.3) | 10.6 (8.4 - 12.8) | 22.9 (19.9 - 25.9) |  |
| Married | 23.2 (20.8 - 25.5) | 20.5 (18.3 - 22.8) | 25.8 (23.4 - 28.2) | 8.0 (6.5 - 9.5) | 22.0 (19.7 - 24.3) | 0.54 |
| Widowed/divorced | 20.9 (12.4 - 29.4) | 19.8 (11.4 - 28.1) | 25.3 (16.2 - 34.4) | 8.8 (2.9 - 14.7) | 24.2 (15.2 - 33.1) | 0.69 |
| Job |  |  |  |  |  |  |
| Office worker | 26.2 (23.6 - 28.8) | 22.0 (19.5 - 24.4) | 23.0 (20.5 - 25.5) | 8.2 (6.6 - 9.8) | 20.3 (17.9 - 22.6) |  |
| Manual worker | 18.9 (13.6 - 24.2) | 15.6 (10.6 - 20.5) | 25.0 (19.1 - 30.9) | 14.6 (9.8 - 19.4) | 25.9 (20.0 - 31.9) | < 0.001 |
| Own business | 18.7 (13.1 - 24.2) | 17.1 (11.7 - 22.5) | 25.9 (19.7 - 32.1) | 10.4 (6.0 - 14.7) | 27.5 (21.1 - 33.8) | 0.001 |
| Housewife/Student/Unemployed | 22.0 (18.6 - 25.4) | 17.4 (14.3 - 20.4) | 28.2 (24.5 - 31.8) | 7.9 (5.7 - 10.1) | 23.5 (20.1 - 27.0) | 0.009 |
| Having a chronic illness |  |  |  |  |  |  |
| No | 25.1 (22.4 - 27.8) | 19.9 (17.4 - 22.3) | 22.9 (20.3 - 25.5) | 8.8 (7.0 - 10.5) | 22.9 (20.3 - 25.5) |  |
| Yes | 22.2 (19.7 - 24.7) | 19.3 (17.0 - 21.7) | 26.7 (24.1 - 29.4) | 9.2 (7.4 - 10.9) | 21.9 (19.5 - 24.4) | 0.46 |

Supplementary Material 2. Factors considered to be important in telemedicine (continued)

| Variable | Selection rate as priority, percent (95%CI) | | | | |  |
| --- | --- | --- | --- | --- | --- | --- |
|  | Possibility of connecting to face-to-face treatment if necessary | Availability whenever the patient needs | Management tailored to each patient's situation and characteristics | Accessibility for use independent of disease type | Availability without economic burden | *P* value |
| Subjective change in health status |  |  |  |  |  |  |
| No change | 23.3 (21.2 - 25.5) | 19.4 (17.4 - 21.4) | 25.2 (23.0 - 27.4) | 8.4 (7.0 - 9.8) | 23.1 (21.0 - 25.3) |  |
| Improved | 27.6 (21.4 - 33.9) | 15.1 (10.1 - 20.1) | 23.6 (17.7 - 29.6) | 14.1 (9.2 - 18.9) | 19.6 (14.0 - 25.2) | 0.61 |
| Worsened | 22.6 (18.6 - 26.6) | 22.4 (18.4 - 26.4) | 24.5 (20.4 - 28.7) | 8.6 (5.9 - 11.3) | 21.2 (17.3 - 25.1) | 0.51 |
| Delayed treatment for chronic conditions |  |  |  |  |  |  |
| No | 22.2 (19.6 - 24.8) | 19.0 (16.5 - 21.5) | 27.7 (24.9 - 30.6) | 8.4 (6.7 - 10.2) | 21.9 (19.3 - 24.5) |  |
| Yes | 22.2 (14.4 - 30.1) | 22.2 (14.4 - 30.1) | 17.6 (10.4 - 24.8) | 15.7 (8.9 - 22.6) | 22.2 (14.4 - 30.1) | 0.74 |
| Delayed elective treatment and treatment for non-chronic conditions |  |  |  |  |  |  |
| No | 23.3 (21.4 - 25.2) | 19.5 (17.7 - 21.2) | 25.3 (23.3 - 27.2) | 8.9 (7.6 - 10.1) | 22.5 (20.7 - 24.4) |  |
| Yes | 27.7 (20.7 - 34.6) | 21.4 (15.0 - 27.8) | 20.1 (13.9 - 26.4) | 10.1 (5.4 - 14.7) | 20.8 (14.5 - 27.1) | 0.28 |
| Experience with telemedicine |  |  |  |  |  |  |
| No | 25.0 (22.8 - 27.2) | 18.5 (16.5 - 20.5) | 26.6 (24.4 - 28.9) | 7.8 (6.4 - 9.1) | 21.5 (19.4 - 23.5) |  |
| Yes | 17.9 (8.7 - 27.1) | 28.4 (17.6 - 39.2) | 19.4 (9.9 - 28.9) | 14.9 (6.4 - 23.5) | 19.4 (9.9 - 28.9) | 0.68 |
